# HOW AN INTERGENERATIONAL TECHNOLOGY INTERNSHIP MODEL BUILDS CAREER READINESS AND AGE FRIENDLINESS

**DOI:** 10.1093/geroni/igad104.1166

**Published:** 2023-12-21

**Authors:** Skye Leedahl, Kristin Souza, Emma Pascuzzi

**Affiliations:** University of Rhode Island, Kingston, Rhode Island, United States; University of Rhode Island, Kingston, Rhode Island, United States; University of Rhode Island, Kingston, Rhode Island, United States

## Abstract

This presentation will describe the key elements of the University of Rhode Island (URI) Engaging Generations Cyber-Seniors Program internship and discuss results from an assessment study that examined post-survey data related to student experiences. Past research has shown that this program is successful in reducing ageism in student participants. In this study, we examined questions from two scales about youth empowerment and participation in planning that corresponded to the Career Readiness Competencies employers seek in graduates today as determined by the National Association of Colleges & Employers (NACE) and measures for building age-friendliness. Survey findings (n=112) showed that the areas in which students stated the most impact were: effort, problem solving, identity exploration, goal setting, and time management. This corresponds to improvements in the NACE Career Readiness Competencies of career & self-development, professionalism, equity & inclusion, and leadership. Related to age-friendliness, scores were particularly high for questions about students speaking out and educating people about older adulthood, telling people about how older people can be digitally included, feeling confident about teaching older adults to use technology, and having ideas about how to improve digital support for older people. The key takeaway from this presentation will be for educators to identify a way in which student experiences can be assessed for growth in career readiness and building age-friendliness.

